# Imaging Mass Cytometry and Single-Cell Genomics Reveal Differential Depletion and Repletion of B-Cell Populations Following Ofatumumab Treatment in Cynomolgus Monkeys

**DOI:** 10.3389/fimmu.2019.01340

**Published:** 2019-06-20

**Authors:** Diethilde Theil, Paul Smith, Catherine Huck, Yoann Gilbart, Algirdas Kakarieka, David Leppert, Celine Rauld, Cindy Schmid, Reto Baumgartner, Nathalie Stuber, Francisco Cordoba, Valerie Dubost, Katy Darribat, Magali Jivkov, Wilfried Frieauff, Rainer Kneuer, Markus Stoeckli, Stefan Reinker, Keith Mansfield, José M. Carballido, Philippe Couttet, Gisbert Weckbecker

**Affiliations:** ^1^Novartis Pharma AG, Basel, Switzerland; ^2^Neurological Clinic and Policlinic, University Hospital Basel, Basel, Switzerland

**Keywords:** B-cell depletion, flow cytometry, lymph nodes, ofatumumab, single-cell genomics, t-SNE, cynomolgus monkeys

## Abstract

Ofatumumab is the first, fully human, anti-CD20 monoclonal antibody in Phase 3 development for multiple sclerosis (MS). The study focused on changes in lymphocyte subsets in blood and lymphoid tissues and on potential novel biomarkers as a result of anti-CD20 antibody action in Cynomolgus monkeys treated with human equivalent doses of subcutaneous (s.c.) ofatumumab on Days 0, 7, and 14. Axillary lymph nodes (LNs) and blood samples were collected at various time points until Day 90. Lymphocyte subsets were quantified by flow cytometry, while morphological and immune cell changes were assessed by imaging mass cytometry (IMC), immunohistochemistry (IHC), in situ hybridization (ISH), and transcriptome analyses using single-cell methodology. Ofatumumab treatment resulted in a potent and rapid reduction of B cells along with a simultaneous drop in CD20^+^ T cell counts. At Day 21, IHC revealed B-cell depletion in the perifollicular and interfollicular area of axillary LNs, while only the core of the germinal center was depleted of CD20^+^CD21^+^ cells. By Day 62, the perifollicular and interfollicular areas were abundantly infiltrated by CD21^+^ B cells and this distribution returned to the baseline cytoarchitecture by Day 90. By IMC CD20^+^CD3^+^CD8^+^ cells could be identified at the margin of the follicles, with a similar pattern of distribution at Day 21 and 90. Single-cell transcriptomics analysis showed that ofatumumab induced reversible changes in t-distributed stochastic neighbor embedding (t-SNE) defined B-cell subsets that may serve as biomarkers for drug action. In summary, low dose s.c. ofatumumab potently depletes both B cells and CD20^+^ T cells but apparently spares marginal zone (MZ) B cells in the spleen and LN. These findings add to our molecular and tissue-architectural understanding of ofatumumab treatment effects on B-cell subsets.

## Introduction

CD20 is a surface membrane-embedded phosphoprotein that is expressed on late pre-B cells, mature B cells and, to some extent, on plasmablasts, but usually not on plasma cells (1). It contributes to the signaling, development and differentiation of B cells but its role is only partially understood. The expression pattern of CD20 is the basis for targeting and thereby depleting B cells. Selective depletion of B cells by anti-CD20 monoclonal antibody (mAb) therapies has been shown to be efficacious in a number of autoimmune diseases including multiple sclerosis (MS) (2–5). These therapies usually involve intravenous (i.v.) administration of high doses of anti-CD20 mAb (4–7), which may not be optimal for selectively targeting disease relevant B cells within lymphatic tissues. Subcutaneous (s.c.) injection is a less intrusive technique for targeting the lymphatic system; s.c. injected antibodies entering the interstitial space follow a decreasing pressure gradient towards lymphatic capillaries and then to the lymph nodes (LNs) (8). Thus, the s.c. route of administration has the potential to target the B cells within the LNs, thereby interfering with autoantibody generation and maturation in the germinal centers of the LNs.

Ofatumumab is the first human anti-CD20 mAb that binds to a composite epitope of CD20 not shared by other anti-MS antibodies. It delivers prolonged and potent depletion activities (9–11), inducing a rapid and efficient elimination of CD20^+^ B cells via complement-dependent cytotoxicity (CDC) and antibody-dependent cell-mediated cytotoxicity (ADCC) (10, 12). Preclinical data show that low-dose s.c. anti-CD20 therapy induces a rapid and efficient B-cell depletion in an MS model (13). Ofatumumab, given by monthly s.c. injections, is being studied in two Phase 3 clinical trials in patients with relapsing-remitting MS (ASCLEPIOS). Low-dose monthly s.c. administration of ofatumumab constitutes a treatment regimen that has been tailored for patients with MS (14).

The present study was undertaken to elucidate the mode of action of s.c. ofatumumab in cynomolgus monkeys, a widely used translational animal model for studying CD20 antibody-based B-cell depleting therapies (15–19). This monkey model allows longitudinal and terminal blood and tissue sampling, enabling a direct comparison of the effects of ofatumumab treatment on B-cell depletion in blood, LNs and the spleen. To better understand the pharmacodynamic effect of ofatumumab in a normal tissue setting, we explored the potential correlations of s.c. ofatumumab-induced changes in lymphocyte subsets in blood and lymphoid tissue by applying state-of-the-art technologies such as molecular imaging by imaging mass cytometry (IMC) and transcriptome analyses by single-cell methodology.

## Materials and Methods

### Experimental Design and Ethical Considerations

A total of 6 cynomolgus monkeys (*Macaca fascicularis*) aged 7–15 years, captive-bred, and with a body weight of 5–9 kg (Siconbrec Inc., Makati City, Philippines) were treated with human equivalent doses of ofatumumab (1 mg/kg) (Supplementary Figure 1). Animals were housed under a maintained temperature (20–24°C), at least 40% humidity and a natural light cycle. All animals were fed at least twice daily with a mixture of fruits or vegetables. Water and Kliba Nafag 3446 pellets (Kaiseraugst, Switzerland) were provided as needed (Supplementary Table 1).

All experimental work on animals was performed in accordance with protocols approved by the Cantonal Veterinary Office of Basel Stadt and according to Swiss Animal Welfare Regulations.

### Drug Administration and Sample Collection

A total of 6 monkeys were treated thrice (Days 0, 7, and 14) with s.c. ofatumumab (100 mg/5 mL, Novartis Pharma AG, Switzerland) administered in the left thigh at a human equivalent dose of 1 mg/kg. In-life LN harvesting was performed a few hours before the first drug administration on Day 0, and on Days 21, 62, and 90 (termination) after the first dose. Axillary LNs were selected and harvested alternatively from the left and right axillar areas, respectively. All remaining axillary LNs were harvested at termination (Day 90 post first treatment). For these short surgical procedures, animals received preemptive analgesia with buprenorphine (0.02 mg/kg intramuscular) and 2 additional doses on the same day of the surgery. Animals were anesthetized with propofol bolus i.v. and maintained as required. More information about these procedures is described in Supplementary section Surgical Procedures.

At the termination of the study (Day 90 post first treatment), animals were euthanized to harvest their spleens and axillary LNs. One part of the spleen was cut into pieces and mechanically dissociated. A single-cell suspension was obtained using 70 μm sieves (Becton Dickinson, USA). Lymphocytes were isolated from whole blood. Details of the preparation of single-cell suspensions are described in Supplementary section Preparation of Single-Cell Suspensions.

Peripheral blood samples were collected from venous blood at baseline (Day 0, a few hours before the first dose) and on Days 2, 9, 16, 21, 28, 42, 62, 78, and 90, which allowed the monitoring of B-cell kinetics during the study. Blood was collected from the cephalic vein into Vacutainer^®^ CPT tubes (Becton Dickinson, USA). After 30 min at room temperature, the CPT tubes were centrifuged for 60 min at 2,900 revolutions per minute (rpm). After 2 washing steps, the peripheral blood mononuclear cells (PBMCs) were resuspended in complete RPMI medium, counted and used either for fluorescence staining or to freeze them down at −80°C in a mixture of 90% of fetal calf serum and 10% of dimethyl-sulfoxide (Sigma-Aldrich, USA) for further analysis.

### Flow Cytometry

Changes in lymphocyte subsets in blood, the spleen and LNs were quantified by Fluorescence-Activated Cell Sorting (FACS) analysis. Multicolor FACS analysis was performed to measure the T cells, B-cell depletion/repletion kinetics. Prior to staining, cells were washed in FACS buffer (PBS containing 2% bovine serum albumin [BSA], 5 mM EDTA), then blocked with a human Fc blocking solution (Biolegend, USA), and stained for 30 min at 4°C with the indicated combination of fluorochrome or biotin conjugated antibodies. When required, a second incubation for 30 min at 4°C was performed with the secondary antibody. Washed and resuspended cells were acquired on a Fortessa flow cytometer (Becton Dickinson, USA). Data were analyzed using FlowJo software (Flow Jo LLC, USA). The flow cytometry antibodies were obtained from commercial sources listed in Supplementary Table 2. The flow cytometry gating strategy is outlined in Table 1.

**Table 1:** Flow cytometry gating strategies and summary of T-cell and B-cell markers.

**Marker/Method**	**Lymphocyte subset/target cell**
**FACS**	
CD20^+^	B cells
CD3^+^ CD20^+^	T cells
CD20^+^ CD268^+^	Activated B cells
**IHC**	
CD21	Follicular and marginal zone B cells
CD1c	Marginal zone B cells, mantle and germinal center B cells
Pax5	Pan B cells
CD3 and CD8	Pan T cells and their subset
**ISH**	
Mfa-CD27	T cells, memory B cells, plasmablast, plasma cells
**IMC (multiplexing/next generation immunohistochemistry)**	
CD3e-170Er, CD8-162Dy, CD20-167Er, CD21-141Pr, Pax5-164Dy, CD163-158Gd, CD68-144Nd, Vimentin-152Sm, Histone H3-176Yb, Iridium intercalator-191Ir, and 193Ir	Panel of T cells, B cells, macrophages markers conjugated to lanthanides

*FACS, Fluorescence-Activated Cell Sorting; Ig, immunoglobulin; IHC, immunohistochemistry; IMC, imaging mass cytometry; ISH, in situ hybridization; Mfa-CD27, Macaca fascicularis CD27 molecule; Pax5, paired box 5 (B-cell specific transcription factor)*.

Parametric unpaired student's *t*-test was performed for analyzing the flow cytometry data using GraphPad Prism (version 4.00; GraphPad Software, San Diego, California, USA). A value of *p* < 0.05 was considered statistically significant.

### Immunohistochemistry (IHC)

IHC and *in situ* hybridization (ISH) were performed for morphological evaluation and quantitative imaging-based immunophenotyping of LNs. IHC staining for all selected markers (Supplementary Table 3) was performed using the fully automated instrument Ventana Discovery XT^®^ or Ventana Discovery^®^ (Roche Diagnostics AG, Rotkreuz, Switzerland). All chemicals were also provided by Roche Diagnostics. Briefly, formalin-fixed, paraffin-embedded tissue sections of 3 μm in thickness were deparaffinized and rehydrated under solvent-free conditions using EZprep™ solution for 8 min at 75°C. Sections were then subjected to heat-induced epitope retrieval by successive cycles in Tris-EDTA based buffer (CC1 solution, option Standard). The slides were blocked using 1x Casein solution in PBS (BioFX laboratories, USA) for 32 min at room temperature to avoid background noise; when necessary, endogenous avidin/biotin activity was quenched by using Ventana A/B blocking reagents (Roche, USA) for 4 min each.

The slides were incubated with the primary antibody for 1–6 h at room temperature. This was followed by a short fixation using 0.05% glutaraldehyde. The slides were then treated with biotin-conjugated or UltraMap anti-rabbit HRP conjugated secondary antibodies. Detection was performed using ChromoMap^®^ kit (Roche, USA) according to the manufacturer's recommendations. The protocol details for each antibody have been summarized in Supplementary Table 4. Counterstaining with Hematoxylin II and Bluing reagent was performed for 2 cycles of 8 min each. Sections were dehydrated and covered using Eukitt (Medite, O1-0500). Stained tissue sections were assessed by light microscopy. Images were captured with the Hamamatsu Nanozoomer slide scanner and Zeiss AxioCam/AxioVision or Aperio.

### *In situ* Hybridization

ISH was performed using the automated instrument Ventana Discovery Ultra^®^ (Roche Diagnostics AG, Rotkreuz, Switzerland). The ISH probes were purchased from Advanced Cell Diagnostics Inc. (Hayward, USA). The PPIB probe was used to measure the RNA integrity and the DapB probe was used as negative control; further details are provided in Supplementary Table 5. All chemicals were provided by either Roche Diagnostics, USA or by Advanced Cell Diagnostics, USA.

Briefly, formalin fixed paraffin embedded sections were deparaffinized manually using 2 baths of xylene for 5 min, followed by 2 baths of ethanol 100% for 1 min, and were then air dried. For the pretreatment steps, slides were immersed in the boiling pretreatment solution (solution Pretreat 2, RNAscope^®^ VS Reagent Kit-RED, Advanced Cell Diagnostics, USA) for 10 min, then refreshed in distilled water at room temperature for 1 min, and finally rinsed in reaction buffer (Reaction buffer, Roche Diagnostics, USA).

Slides were placed in the Ventana Ultra instrument and started using the procedure mRNA Red discovery Ultra 4.0 with the predefined parameters and using the combined Ventana and Advanced Cell Diagnostics required kit reagents (RNAscope^®^ VS Reagent Kit-RED, and mRNA RED, Amp & Pretreatment PTO kit). Counterstaining was performed using Hematoxylin II for 8 min followed by Bluing reagent for 8 min. Sections were mounted in glycerol-gelatin mounting medium (Sigma-Aldrich Chemie GmbH, Buchs, Switzerland, reference GG1) and dried on a hot plate at 42°C for at least 1 h before microscopic examination. Stained tissue sections were assessed by light microscopy. The slides were scanned on the NanoZoomer 2.0-HT scanner instrument (Hamamatsu Photonics France, Massy, France) and/or Zeiss AxioCam/AxioVision using the x40 objective.

### Quantitative Image Analysis

IHC stained LN sections (of 3 μm thickness) were scanned with Aperio's ScanScope scanner at 20 × magnification. The ImageScope software (V12.1.0.5029, Aperio Inc., USA) with its interactive pen tool for outlining regions of interest was used to manually draw annotation outlines to identify follicles for the quantification of CD1c, CD21, CD8, and Pax5 expression assessment in axillary LNs, deemed suitable for subsequent image analysis. The resulting annotation file (.xml format) was then used for the creation and export of ^*^.tif images, using an in-house developed ImageScope plug-in. Image tiles were created for the region annotations and stored accordingly.

Customized modules to quantify the numerical distribution of marker-positive and marker-negative cells within the specified region of interest were developed as integration in a proprietary image analysis platform (ASTORIA, Automated Stored Image Analysis), running under MS Visual Studio 2010 and based on many tool functions and algorithms using Matrox MIL V9 libraries (Matrox Inc., Quebec, Canada). No detailed listing or specification of the functions and modules is given since the Matrox MIL libraries consist of hundreds of low-level functions serving as a basis for more complex tool modules, mostly in the realm of Mathematical Morphology, which have been developed through the years. The sequential steps followed for image analyses are described in Supplementary section Sequential Steps for Image Analysis.

### Imaging Mass Cytometry

Imaging mass cytometry was used for the detection of multiple markers on the same tissue section. A panel of 9 antibodies to identify B cells, T cells and macrophage cell types was used by IMC. The target, provider, clone, and metal tag of each antibody used in this study are listed in Supplementary Table 6. Metal tag selection for each antibody was done using the interactive web-based Maxpar^®^ Panel Designer tool. Antibody conjugations were performed using the Maxpar^®^ X8 Antibody Labeling Kit (Fluidigm Sciences Inc., USA) according to the manufacturer's instructions. After labeling, the concentration of each antibody was determined using a Nanodrop UV-Vis Spectrophotometer (Thermo Scientific, USA). Formalin fixed paraffin embedded axillary LN tissue sections of 5 μm in thickness were dewaxed and rehydrated in standard conditions. Heat-induced epitope retrieval was conducted in a bath at 95°C of Target Retrieval Solution (Dako Agilent Technologies, USA) for 20 min. After immediate cooling, the sections were blocked with 1% BSA (Miltenyi Biotec Ltd., Germany) and 0.1% TritonX-100 (Sigma-Aldrich Chemie, Switzerland) diluted in PBS for 30 min. Samples were incubated overnight at 4°C with 200 μL of the antibody cocktail diluted in 1% BSA and 0.1% TritonX-100 in PBS. The concentration of each antibody varied between 0.5 and 2 μg per section. After incubation, a short post-fixation with paraformaldehyde 4% was done before exposure to 125 μM Cell-ID Intercalator-Ir (Fluidigm Sciences Inc., USA). Tissue sections were rinsed twice in distilled water and air dried at room temperature.

Samples were introduced in a prototype laser ablation source (beta of commercial system “Hyperion” from Fluidigm, Markham, CN) attached to an ICP mass analyzer (“Helios” from Fluidigm, Markham, CN). After flushing the ablation chamber with Helium, the sample areas were ablated at a raster of 1 μm with a speed of 200 pixels/s. The signal for each marker was captured using proprietary software and the data was assembled in OME.tiff files for further processing.

The obtained multilayer TIFF images were pre-processed in the statistical software R for de-noising and quality control. An average DNA image was produced by summing the 2 DNA channels Ir191 and Ir193, and the resulting nuclear stain image was segmented in CellProfiler software using Otsu's method of local thresholding. Subsequently, to define the cytoplasm, a Voronoi expansion was used, and for each channel the integrated intensity over the cell area was computed. The resulting table of all cells from an image including location and protein expression was loaded again into Spotfire for gating and population analysis.

### Single-Cell Targeted Gene Expression Analysis

Targeted gene expression profiling was performed on CD20^+^/CD3^−^ FACS-sorted B cells from axillary LNs at the single-cell level at different time points during the ofatumumab treatment (Days 0, 21, 62, and 90). The viability of CD20^+^/CD3^−^ cells was confirmed using a LIVE/DEAD fixable Aqua cell stain kit (Thermofisher, USA, reference L34957). Fluidigm integrated fluidic circuits (IFCs) from the same batch were used to capture single cells of small sizes: 5–10 μm (B cells). Captured cells were imaged on chips using a Julistage microscope (NanoEntek, USA) to confirm the number of cells per site. Only single cells were considered for further analysis.

Capture, cell lysis, cDNA synthesis and pre-amplification were performed using Fluidigm's C1 Single-Cell Auto Prep System (Fluidigm Sciences Inc., USA). Commercially available Taqman assays (Thermofisher, USA) were purchased for the gene of interest that included a specific set of signatures indicative of every B-cell subset, of general T cells, and of proliferation (Supplementary Table 7). Single-cell targeted gene expression profiling of CD20^+^/CD3^−^ cells was carried out using automated, high-throughput quantitative polymerase chain reaction (PCR) analysis using 96.96 dynamic array IFCs on the BioMark HD System (Fluidigm Sciences Inc., USA). Raw data for gene expression were obtained as threshold cycle (C_t_) values and normalized following the delta Ct (dCt) method. Delta Ct values were calculated for every gene following the formula: dCt = Ct gene of interest – Average (Ct 18S RNA + Ct β-actin). Linear values of gene levels were obtained by calculating 2^−dCt^. Expression values were then transformed in log10 and uploaded into R/Bioconductor. A dimensionality reduction algorithm, known as t-distributed stochastic neighbor embedding (t-SNE), was then applied to the 19 dynamic arrays to facilitate the identification of similar cells. Tibco-Spotifire software was then used to visualize t-SNE coordinates.

## Results

### Changes in Lymphocyte Subsets in Blood in Response to Subcutaneous Ofatumumab Treatment in Cynomolgus Monkeys

**Figure 1:**
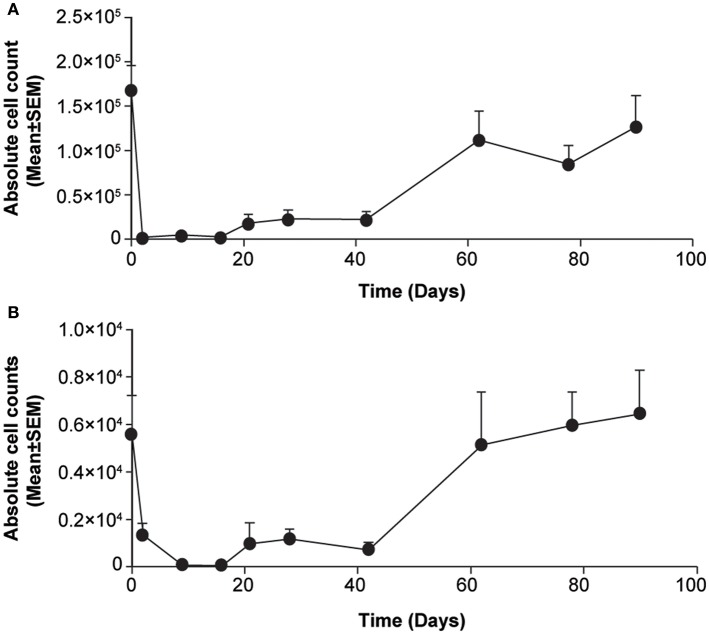
Changes in lymphocyte counts in blood samples from cynomolgus monkeys treated acutely with subcutaneous ofatumumab. **(A)** CD20^+^ B cells. **(B)** CD3^+^CD20^+^ T cells. Data are expressed as means ± SEM. SEM, standard error of the mean.

Treatment with s.c. ofatumumab resulted in a rapid and strong depletion of total B cells (CD20^+^) as early as Day 2, with B-cell counts being decreased still on Day 30 by more than 80% against baseline. The repletion was apparently biphasic in that the initially moderate repletion started on Day 21 and accelerated beyond Day 40 (Figure 1A). A subset of T cells (CD3^+^ CD20^+^) showed similar depletion and repletion kinetics (Figure 1B). All depleted B-cell subsets were also B-cell activating factor receptor (BAFF-R, also referred to as CD268) positive. Throughout the observation period, the number of CD20^+^ B cells was very similar to the number of B cells (CD20^+^CD268^+^; Supplementary Figure 2). Overall, the treatment was well-tolerated by these animals, with no local or systemic adverse effects.

### Changes in Lymphocyte Subsets in Axillary LNs Assessed by IHC

**Figure 2:**
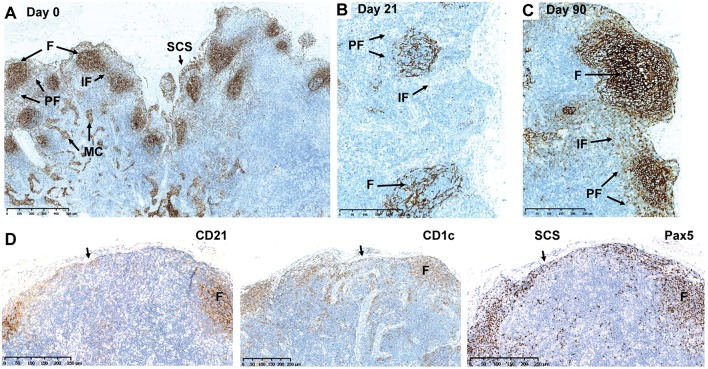
Depletion and repletion of B cells in the axillary LNs after subcutaneous ofatumumab treatment shown by CD21, CD1c, and Pax5 IHC. **(A)** Normal distribution of CD21^+^ B cells before treatment. **(B)** Depletion of CD21^+^ B cells at Day 21. **(C)** Repletion of CD21^+^ B cells at Day 90. **(D)** Comparison of the CD21, CD1c, and Pax IHC in the same axillary LN during depletion (Day 21). F, follicles; IF, interfollicular region; IHC, immunohistochemistry; LN, lymph node; MC, medullary cords; Pax5, paired box 5; PF, perifollicular region; SCS, subcapsular sinus.

IHC was applied to investigate B-cell subsets in their respective anatomical regions of axillary LNs from monkeys treated by s.c. ofatumumab. B-cell subsets were differentially impacted, as evidenced by the distribution of CD21^+^ B cells (follicular and marginal zone B cells) before and after s.c. ofatumumab treatment (Figure 2). At Day 21, complete depletion of CD21^+^ B cells was observed in the perifollicular and interfollicular areas of the axillary LNs. Less severe depletion was observed in the lymphoid follicles (Figure 2B). At Day 62, the repletion of CD21^+^ B cells was prominent, showing abundant infiltration into the perifollicular and interfollicular regions. At Day 90, the repletion was complete, as indicated by the normal distribution of CD21^+^ B cells observed in the follicular and perifollicular areas (Figure 2C). These results were further confirmed by quantitative imaging of CD21^+^ staining in the axillary LN sections on Day 21 and Day 90 (Supplementary Figure 3). In parallel with the CD21 staining, adjacent LN sections were stained with CD1c and Pax5 and showed a similar pattern overall compared to CD21. Comparison of the staining patterns of the latter B-cell markers at Day 21 revealed the presence of B cells in the follicles and in the subcapsular sinus of the axillary LNs (Figure 2D, arrows).

**Figure 3:**
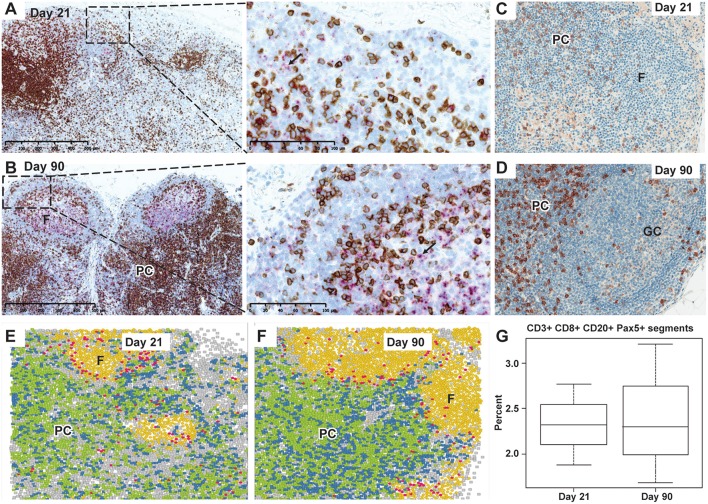
Depletion and repletion of CD27^+^ cells and CD3^+^/CD8^+^ T cells and representation of CD20^+^CD3^+^CD8^+^ cells by IMC in the axillary lymph nodes after subcutaneous ofatumumab treatment. **(A)** Depletion in CD3^+^ T cells (brown) in the PC and CD27 expressing B cells (pink) in the follicles at Day 21. **(B)** Normal lymph node cytoarchitecture showing secondary B-cell follicles with germinal center formation (pink, CD27 ISH) and CD3^+^ T-cell repletion (brown) at Day 90 (dual CD3 IHC and CD27 ISH). **(C)** Depletion in CD8^+^ T cells in the PC at Day 21. **(D)** Repletion of CD8^+^ T cells (brown) in the PC at Day 90. **(E)** Identification and distribution of CD20^+^CD3^+^CD8^+^ cells (IMC, pink) around the B-cell follicles at Day 21. **(F)** Identification and distribution of CD20^+^CD3^+^CD8^+^ cells (IMC, pink) around the B-cell follicles at Day 90. **(G)** Quantitative imaging of a subset of CD20^+^CD3^+^CD8^+^ cells, similar at Day 21 and Day 90 and confirmed to be Pax5 positive. IMC data reconstruction after laser ablation of lymph node tissue section and color assignment to multiple positive cells: • Background; • CD3^+^ CD8^−^; • CD3^+^ CD8^+^; • CD20^+^; • CD20^+^ CD3^+^ CD8^+^; F, follicles; GC, germinal center; IMC, imaging mass cytometry; Pax5, paired box 5; PC, para cortex.

Changes in T cells were also observed in axillary LNs when assessed using a combination of CD3 IHC and CD27 ISH or single CD8 IHC. A depletion of CD3^+^ CD8^+^ T cells at Day 21 was followed by a repletion at Day 90 (Figures 3A–D). By using IMC, we identified the rare CD3^+^CD8^+^CD20^+^ T-cell subpopulation, localized in the periphery of the B-cell follicles, which may additionally express Pax5. No apparent difference was seen in this subpopulation at Days 21 and 90 regarding cell frequency and anatomical distribution (Figures 3E–G).

The inter-individual variability in terms of the image analyses has been provided in the Supplementary Table 8.

### Changes in Lymphocyte Subsets in the Spleen

**Figure 4:**
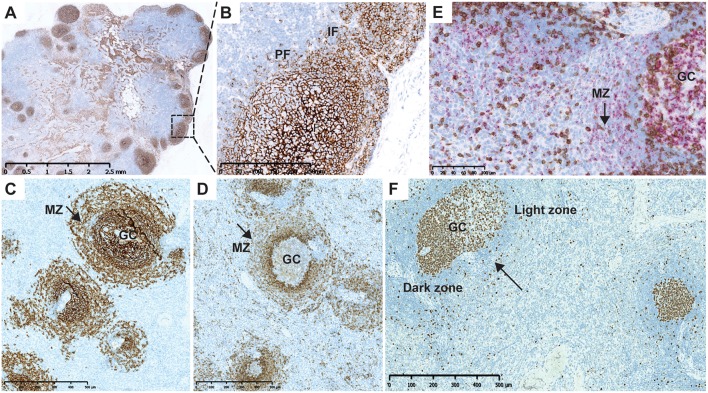
Cytoarchitecture of the lymph node and spleen after repletion at Day 90. **(A)** CD21 IHC (brown) illustrating normal cytoarchitectural organization of the lymph node at Day 90. **(B)** Higher magnification of the region marked in **(A)** (rectangle). CD21^+^ B cells populating the entire follicle and subcapsular sinus with dissemination in the IF and PF. **(C)** Normal B-cell distribution in the follicles and MZ of the spleen after ofatumumab treatment. CD21 (F and MZ) IHC. **(D)** Normal marginal zone B-cell distribution after treatment with ofatumumab; CD1c (MZ marker, PF) IHC showing the MZ. **(E)** CD3 IHC (brown) and CD27 ISH (pink) after treatment with ofatumumab illustrating normal distribution of CD27^+^ B cells in the GC and MZ (dual CD3 IHC and CD27 ISH). **(F)** Ki67 staining in the spleen showing proliferation in the core of follicles (arrow points to a secondary follicle with a mature GC). F, follicles; GC, germinal center; IF, interfollicular region; IHC, immunohistochemistry; PF, perifollicular region, MZ, marginal zone.

A normal cytoarchitectural organization of the spleen was observed at Day 90, the day of necropsy, as confirmed by CD21 (Figures 4A,B) and CD1c IHC (Figures 4C,D). Normal distribution of CD27^+^ B cells was observed in the germinal center and marginal B-cell zone of the spleen in ofatumumab-treated monkeys (Figure 4E) when compared to control tissue (i.e., the spleen of a healthy monkey).

In the course of the image analysis assessment, follicles were semi-automatically assessed in the axillary LNs, based on the CD8, and CD21 staining. These results are in line with the quantification of CD8 and CD21, showing depletion in both cell populations. A more detailed assessment of the status of the lymph follicle was done in the spleen and was based on the Ki67 IHC, as GCs in macaques can be readily identified as positive for the B cell marker CD20 and express high levels of the proliferation marker Ki67 (CD20pos Ki67hi) (20). Mature secondary germinal center were identified based on the size and segregation in the dark and light zones, easily recognizable based on the Ki67 staining pattern (Figure 4F, arrow) (21). The percentage of secondary follicles with mature germinal centers are presented in Supplementary Figure 5.

### t-SNE Analysis of CD20^+^ B Cells Identifies Time-Dependent B-Cell Cluster Formation

**Figure 5:**
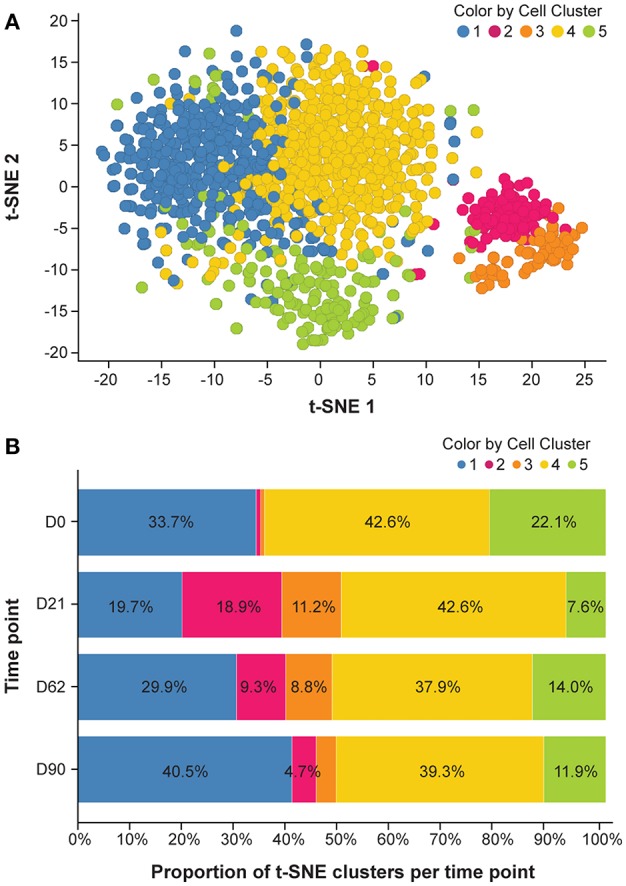
mRNA expression analysis. **(A)** Cluster of t-SNE across all time points. Analysis was done to reduce data dimensionality and facilitate the identification of similar cells. **(B)** Dynamic aspect of ofatumumab treatment. Please refer to the Supplementary Table 7 for details of individual clusters. D, day; t-SNE, t-distributed stochastic neighbor embedding.

To explore changes of key molecules defining B-cell category and function, we performed single-cell transcript analysis of LN cells harvested on Days 0, 21, 62, and 90. A total of 1,292 CD20^+^/CD3^−^ individual cells were isolated from axillary LNs of 6 animals and more than 80 mRNA markers were detected in all cells. Analysis of global mRNA expression levels in these 1,292 cells showed 5 t-SNE-defined cell clusters in untreated monkeys (Figure 5A). t-SNE clusters 1 to 5 across all time points are presented in Figure 5B. Treatment with ofatumumab showed an increase of the proportion of clusters 2 and 3 that were very small at baseline, increased at Day 21 and started to return to baseline by Day 62. Inversely, the treatment with ofatumumab also showed a decrease of the proportion of clusters 1 and 5 and did not affect the proportion of cluster 4. The percentage of cells expressing a gene of interest was calculated per cluster (Supplementary Table 9). Data were submitted to a hierarchical clustering in 2 dimensions to phenotype each cluster (Supplementary Figure 4). All of the 1,292 cells analyzed expressed known B-cell markers such as CD20, CD19, CD79A/B, CD1c, and CD45 along with markers of cell function and activation such as CD74, CD289-TLR9, HDAC7, BCL6, CD282-TLR2, TLR7, and CD24 mRNAs. The expression patterns of mRNA of clusters 2 and 3 showed certain similarities. Most cells expressed AID, AICDA, CD95-FAS, FOXO1, CD275, and TCF3 mRNAs. Moreover, the majority of cluster 2 and 3 B cells expressed proliferation markers such as MKI67, RRM2, CCNB1, CCNA2, and TOP2A mRNAs. These gene signatures suggested that B cells from cluster 2 may be part of the germinal center and cluster 3 B cells may have a memory phenotype. The mRNA content of clusters 1 and 4 was similar, in that most cells expressed CD62L, CD49, and CD38 mRNAs. However, most cluster 4 B cells (but not cluster 1) expressed markers such as TFE3, CD86, CD80, and CD29 mRNAs, suggesting that cluster 1 might be assigned as transitional B cells and cluster 4 as activated B cells. Finally, most cells from cluster 5 expressed BLIMP1-PRD, TOP2A, and CD21 mRNAs and were negative for CD19 and CD81 mRNAs, suggesting a plasma cell phenotype.

## Discussion

The present study on the effects of ofatumumab is the first detailed *in situ* characterization of B-cell subsets in non-human primates (NHP) after s.c. administration of an anti-CD20 depleting antibody at a human-equivalent therapeutic dose. By combining state-of-the-art localization techniques such as IHC, ISH and highly multiplexed imaging and single-cell genomics, we demonstrated that the B cells located in the perifollicular and interfollicular spaces (CD20^+^CD21^+^) are completely depleted in the axillary LNs, which was chosen to represent non-draining LNs relative to the s.c. injection site. No molecular changes were found in the perifollicular zone of the spleen. Residual B cells sharing the same phenotype (CD21^+^ CD1c^+^) were found in the follicle and subcapsular space. Ki67 staining patterns in these areas suggested that part of these cells are newly (locally) generated cells. The number and size of the follicles in the LNs and spleen at Day 90 varied among the individual animals and was in line with published data from vehicle treated cynomolgus monkey or recovery animals after treatment with the anti-CD40 antibody (22, 23).

The current study was performed in healthy monkeys. We cannot exclude that results would be somewhat different in monkeys undergoing an inflammatory attack mimicking MS. Therefore, it may be considered to extend the work reported here by future studies that focus on the effects of ofatumumab e.g., in a monkey EAE model (24). Such studies may include a comparison of s.c. vs. i.v. administration of the anti-CD20 antibodies as well as distribution of the anti-CD20 antibodies in blood, lymphoid tissue and brain to further understand the potential advantages of s.c. administration over the i.v. route.

Low-dose s.c. ofatumumab treatment effectively targeted lymphocytes within the LNs and induced reversible changes in B-cell subsets. B-cell depletion in the lymphoid tissue was accompanied by a reduction of T cells. However, the subset of CD3^+^ CD20^+^ T cells in the axillary lymph node was similar at Day 21 and Day 90. By contrast, in the NHP blood, CD3^+^ CD20^+^ T cells followed the same depletion and repletion kinetics as B cells. Palanichamy et al. (25) demonstrated that treatment of MS patients with high dose rituximab given by infusion led to a decrease in blood CD20^+^ T cells. The authors speculated that this T cell subset may occur at an early stage of T cell development which may explain its long-term depletion under rituximab. This study did not provide data on changes of CD20^+^ T cells in tissue. While i.v. infused ocrelizumab in MS patients depleted blood CD20^+^ T cells, effects on tissue CD20^+^ T cells were not reported (26). Interestingly, previous studies showed that CD3^+^ CD20^+^ T cells apparently display an activated phenotype (27). A positive correlation between disease severity and CD20^+^ T cells in CSF in patients with MS, was recently reported (28). However, there is limited understanding regarding the role of CD20^+^ T cells in MS pathogenesis. Accordingly, our knowledge of differential effects of various anti-CD20 antibodies and various routes of administration and doses on CD20^+^ T cell levels in blood and tissue is still incomplete. While the cynomolgus study reported here sheds further light on blood and tissue CD20^+^ T cells, it is obvious that more in depth molecular investigations are needed to elucidate the function, location and dynamics of this immune cell subset.

Our molecular investigations in the monkey show, that B-cell subsets or B cells homing to specific anatomical regions show different susceptibility to ofatumumab depletion. These findings are in line with previous studies in monkey lymphoid tissue with the other anti-CD20 antibodies, which showed different susceptibility of B cells (15, 16, 29). The different susceptibility of B-cell subtypes may be linked to the bioavailability of the antibody through the lymphatic system after s.c. injection, or may be related to the phenotypic makeup in these specific anatomic regions (30).

The presence of BAFF-R on apparently all B cells during the maximal depletion phase may be important for B-cell survival and efficient repletion. Relatively, the marginal zone B-cell subsets residing in the subcapsular sinus are spared from ofatumumab-induced depletion. This subset is functionally important in immune defense against blood-borne bacterial and viral infections (31, 32). Proliferation of the persisting resident B cells (marginal zone and nodal B cells) suggests that these B-cell types have backup functions during the depletion phase. These cells are not only the gatekeepers of the innate immunity but may also support quick recovery due to cytokine or surviving factor production (33, 34). The augmented B-cell clusters detected by single-cell genomics may reflect a proliferation/repletion from the resident pool. Comparison of single-cell genomics from the blood will help to elucidate this.

Subcutaneous administration of ofatumumab induced reversible changes in B-cell subsets in this NHP model. The normal appearance of the cytoarchitectural organization of LNs and the spleen at Day 90 suggests recovery and normal functioning of the secondary lymphoid system after depletion with ofatumumab. The extent of depletion in blood and tissue, after administration of 3 low doses of s.c. ofatumumab in monkeys, was comparable to the depletion achieved with high doses of other CD20 antibody types in monkeys (16, 18).

A more rapid B-cell repletion after s.c. ofatumumab may be ascribed to a better cytoarchitectural preservation of anatomical sites with regenerative properties, and thus ensures better B-cell homeostasis.

Single-cell mRNA expression analysis in small-size samples is a new approach for identifying B-cell subsets, independent of known marker sets. We analyzed single-cell mRNA content by applying a dimensionality reduction algorithm called t-SNE to determine the effect of ofatumumab treatment on mRNA expression patterns in B cells isolated from LNs over time. The dynamic aspect of this analysis, in line with molecular localization in lymphoid tissue, gave us an unprecedented description of the reversible ofatumumab effect and therefore a better understanding of the treatment effect on B-cell subsets. The visualization of single-cell transcriptomics data by looking at the proportion of cells expressing a particular gene per cluster helped us to define gene signatures to each cluster. These gene signatures were then compared to literature to propose a B-cell subset phenotype to each cluster. Notably, it is also possible to cluster cells in a t-SNE-independent manner to identify new B-cell subsets. The analysis of larger numbers of individual cells and also more genes, e.g., by use of RefSeq, might help us to better assign B-cell phenotypes to clusters. The initial single-cell analysis data provided here suggest the existence of ofatumumab-sensitive t-SNE-defined clusters that may serve as a starting point for the definition of novel biomarkers responsive to B cell-targeting treatments.

In conclusion, in s.c. ofatumumab-treated monkeys, we showed that the combination of single-cell transcriptomics with novel molecular localization techniques such as IMC improved our molecular understanding of the effects of B-cell depleting therapies on B-cell subsets and suggested potential new pharmacodynamic biomarkers.

## Data Availability

All single cell genomics data (t-SNE coordinates, normalized expression values) have been included in the Supplementary Material.

## Ethics Statement

All experimental work on animals was performed in accordance with protocols approved by the Cantonal Veterinary Office of Basel Stadt and according to Swiss Animal Welfare Regulations.

## Author Contributions

DT and KM led the molecular localization investigations and interpretation. CH, FC, NS, RB, CR, JC, and CS were involved in the experimental design, methodology and analysis of flow cytometry, and performance of the in-life study. KD was involved in the performance of the *in situ* hybridization and immunohistochemistry. YG has been instrumental in performing the single cell analysis. WF was involved in the performance of the digital image analysis. PS was the overall project lead and involved in the experimental design, methodology, and analysis of flow cytometry. He worked on obtaining the animal license/ethical approvals. VD and MS were involved in experimental design and data interpretation of imaging mass cytometry. RK was involved in labeling the imaging mass cytometry antibodies. MJ and SR performed the analysis of the imaging mass cytometry experiment. JC, DL, and AK were involved in the drug development activities. PC was involved in the design and data analysis of single-cell genomics. GW led significant activities of data compilation and interpretation and co-developed and edited the manuscript.

### Conflict of Interest Statement

This study was funded by Novartis Pharma AG, Basel, Switzerland. The study sponsor participated in the design and conduct of the study, data collection, data management, data analysis and interpretation, and preparation, review, and approval of the manuscript. All authors are employees of Novartis Pharma AG, Basel. Paul Smith and David Leppert were employees of Novartis at the time of the conduct of the study.

## Abbreviations

ADCC: antibody-dependent cell-mediated cytotoxicity
CDC: complement-dependent cytotoxicity
C_t_: threshold cycle
FACS: fluorescence-activated cell sorting
IFC: integrated fluidic circuits
IHC: immunohistochemistry
IMC: imaging mass cytometry
ISH: *in situ* hybridization
i.v.: intravenous
LN: lymph nodes
mAb: monoclonal antibody
MS: multiple sclerosis
NHP: non-human primates
PBMC: peripheral blood mononuclear cells
PCR: polymerase chain reaction
rpm: revolutions per minute
s.c.: subcutaneous
t-SNE: t-distributed stochastic neighbor embedding.
