# Supplementary material for: Imaging Mass Cytometry and Single-Cell Genomics Reveal Differential Depletion and Repletion of B-Cell Populations Following Ofatumumab Treatment in Cynomolgus Monkeys
Source: Front Immunol. 2019 Jun 20;10:1340. doi: 10.3389/fimmu.2019.01340 (PMC6596277; doi:10.3389/fimmu.2019.01340)
Supplement: Supplementary file 1 [file Data_Sheet_1.pdf]

SUPPLEMENTARY MATERIALS

1.0 Surgical procedures

Animals were anesthetized with intravenous (i.v.) propofol bolus and maintained as required. Once the animals were immobilized, body weights were recorded and hair from the surgical area was trimmed. Consecutively, animals received medical oxygen, with heart rate, blood pressure and oxygen saturation monitored. The skin was disinfected with Cutasept® (IVF Hartmann AG, Germany) and approximatively 5 cm incisions were made. LNs were exposed by atraumatic dissection of the adipose tissue and harvested using vascular ligation whenever necessary. Finally, subcutaneous (s.c.) tissue and skin were sutured separately using PDSII 5/0 (Braun, Germany) and disinfected locally with chlorhexidine (Bepanthene Spray®, Bayer, Germany).

2.0 Preparation of single-cell suspensions

At each time point, at least one lymph node (LN) was used to prepare single-cell suspensions by mechanically dissociating the tissue into ice-cold Roswell Park Memorial Institute (RPMI) 1640 growth medium (Life Technologies, USA) containing 10% of heat-inactivated fetal calf serum (Bioconcept, Switzerland), 50 nM of beta-mercaptoethanol and penicillin/streptomycin (Life Technologies, USA). These suspensions were stored at −80ºC in a mixture of 90% fetal calf serum and 10% dimethyl-sulfoxide (Sigma-Aldrich, USA) until processed further. At least one LN was formalin fixed and paraffin embedded for immunohistochemical investigations.

3.0 Sequential steps for image analysis

For the image analysis of individual *.tif files, each containing one of the selected follicles, the following sequence of steps was performed.

1. For the CD1c and CD21 expression quantification (no individual cell segmentation and cell count possible, thus, only marker-positive area ratio was used as the readout):
    - Color deconvolution
    - Determination of stained area within follicle based on average gray value (red channel of the image)
    - Assessment of brown stained area percentage based on gray mean in the blue channel of the color-deconvolved image
    - Morphological filtering (opening, closing, size filters)
    - Numerical result collection (values for blue, brown and total stained area)
2. For CD8 and Pax5 analysis (segmentation of individual nuclei for the analysis as well as marker-positive area ratio):
    - Color deconvolution
    - Edge detection and hole filling with morphological filtering for a first pass to collect valid nuclei
    - Iterated gradient + watershed approaches for subsequent segmentation to refine nuclear detection and collection (iteration through contrast thresholds, resulting in collecting sufficiently round nuclei within acceptable size limits)

- Similar segmentation looping like before, this time using the tophat transformation with adaptive thresholding instead of gradient + watershed transformation
- Pooling of all objects that were segmented by the previous algorithms
- Removal of artificial debris and regions that are too dark
- Post-processing of segmented nuclei (filtering to separate invalid objects, classification of valid into blue and brown classes based on intrinsic gray statistics of deconvolution images)
- Numerical result collection (values for blue, brown and total stained area as well as individual cell counts)

For details on the theoretical background of transformations using the concept of Mathematical Morphology, such as gradient-watershed or tophat transformation, please refer to.[1,2] Some variation in quality necessitated rather complex and sophisticated segmentation methodology.

References:

1. Serra J, Soille P: Mathematical Morphology and its Applications to Image Processing. Dordrecht-Boston-London.: Kluwer Academic Publishers, 1994
2. Sternberg SR: Grayscale morphology. Comput Vision Graph Image Process 1986, 35:333-355

Supplementary Figure 1. Drug administration and sample collection

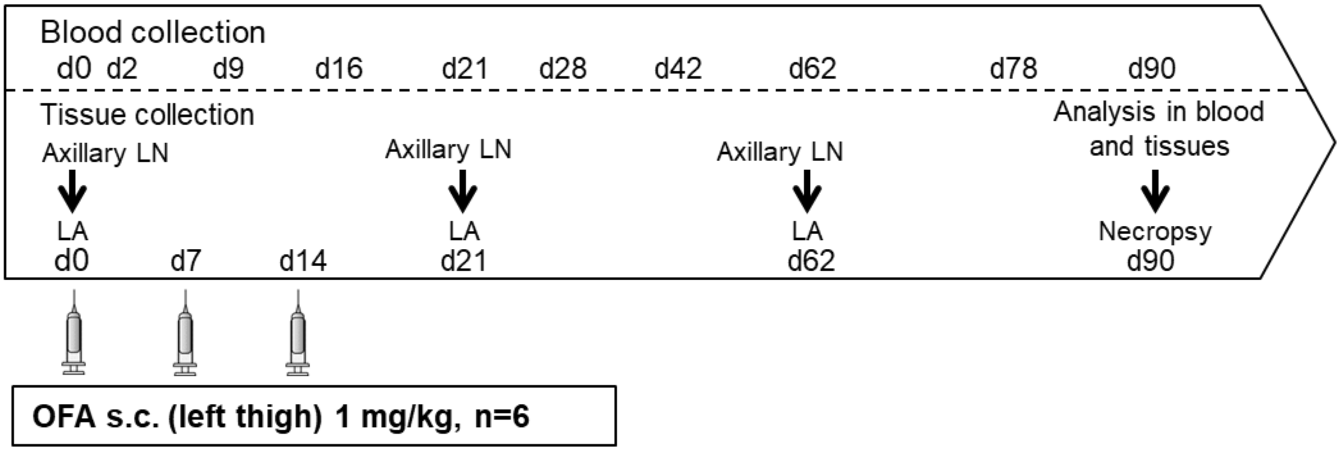

d, day; LA, lymphadenectomy; LN, lymph node; OFA, ofatumumab; s.c., subcutaneous

Supplementary Figure 2. Changes in lymphocyte counts (CD20+ CD268+ cells) in blood

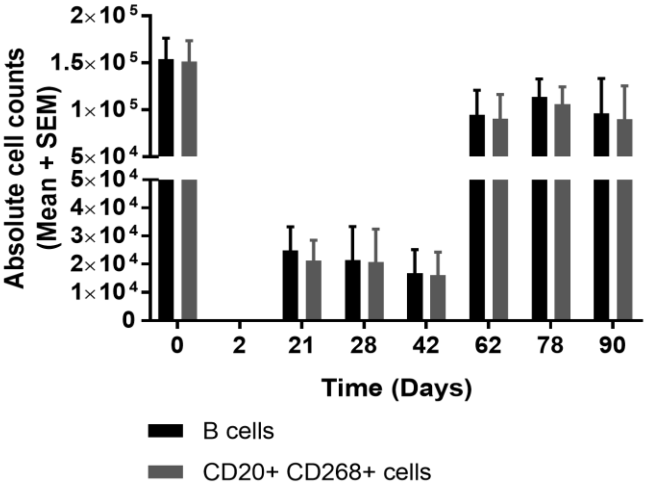

SEM, standard error of the mean

Supplementary Figure 3. Quantitative imaging of CD21+ staining in axillary LN sections at Day 21 and Day 90.

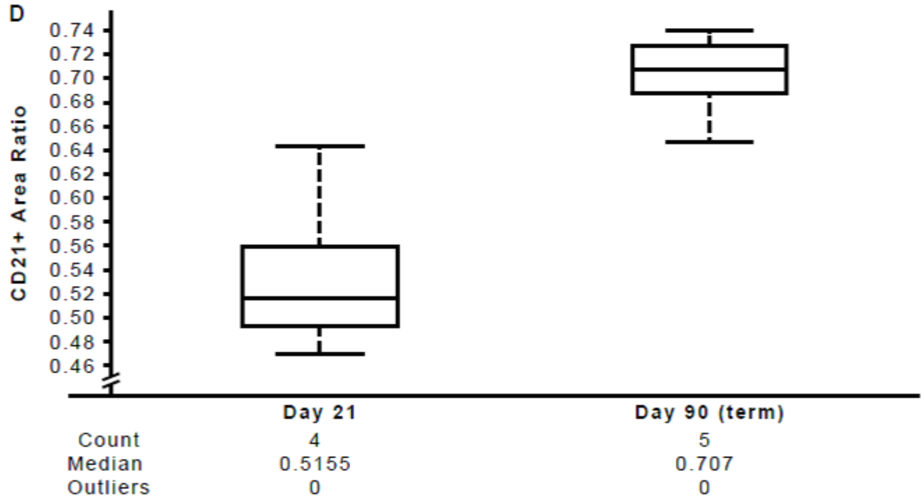

Time points (Day 21 and 90) are represented on the X-axis. The ratio of CD21 stained:unstained tissue is represented on the Y-axis.

Supplementary Figure 4. Hierarchical clustering

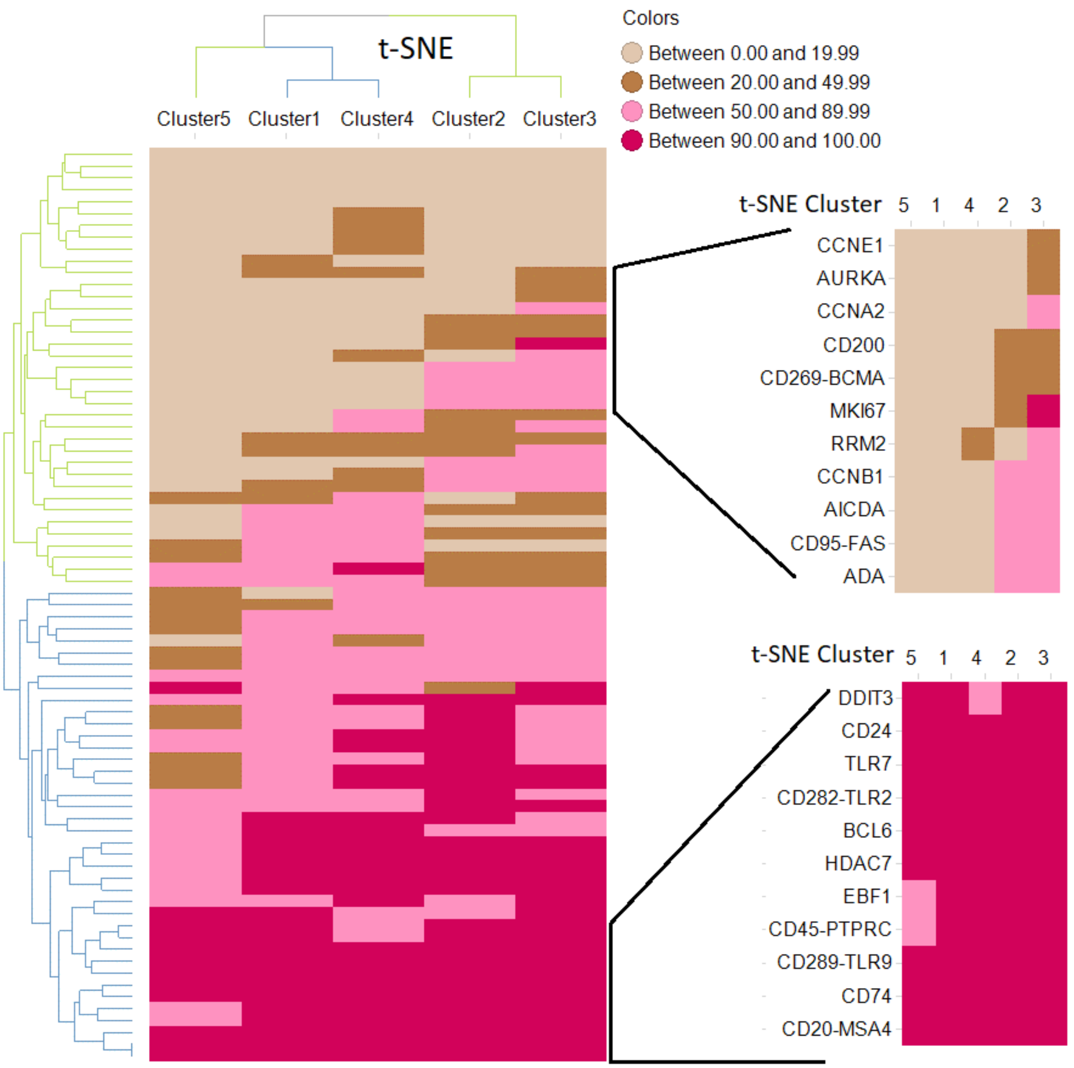

t-SNE, t-distributed stochastic neighbor embedding

Supplementary figure 5. Percentage of secondary follicles as assessed by Ki67 staining

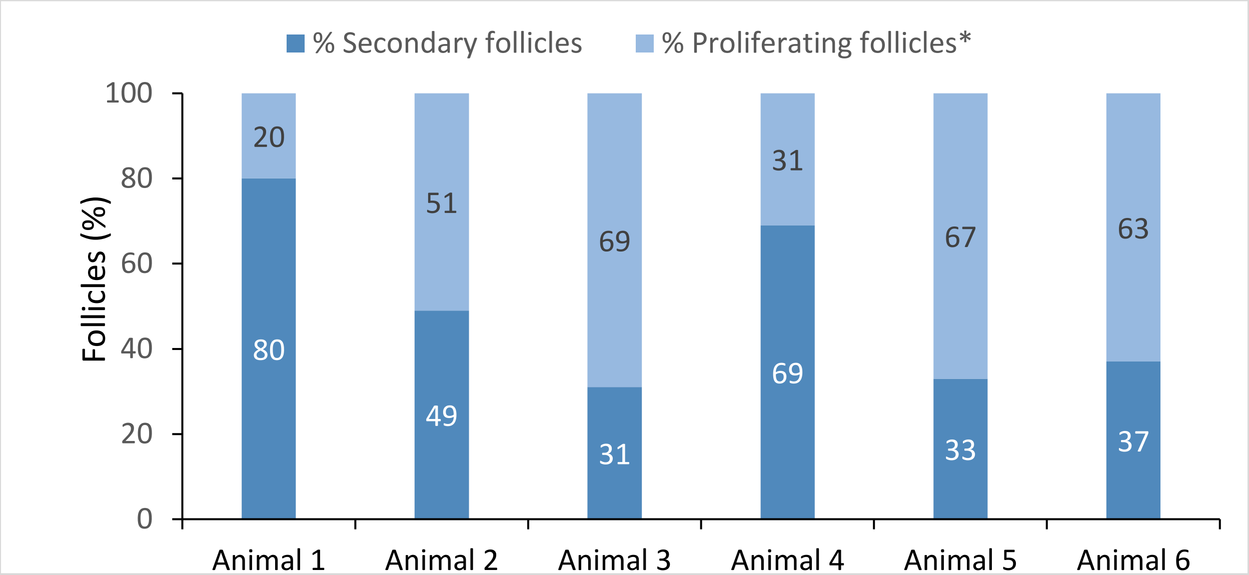

*Include secondary follicles with early germinal center

Supplementary Table 1. Animal housing

<table>
<tr><td>Cyno source</td><td colspan="4">SICONBREC, INC 6th Floor Kings Court I 2129 Pasong Tamo St, Makati City, Philippines</td></tr>
<tr><td>Housing</td><td colspan="4">Males and females were housed separately in groups. Animals were isolated in case of incompatibility/aggressiveness. All animals had access to the large pen 20+ hours per day.</td></tr>
<tr><td>Bedding</td><td colspan="4">Woodchip bedding: Rettenmeier Granulat M8-15 (Wilburgstetten, Germany)</td></tr>
<tr><td>Fresh food</td><td colspan="4">Seasonal Vegetable and Fruits (Source Marktsteiner Switzerland). Vegetable once daily and fruits twice weekly.</td></tr>
<tr><td>Pellets</td><td colspan="4">100g/animal/day; NAFAG-Kliba Primate Extrudat High Fiber Nr. 3496, Pellet 12 mm round</td></tr>
<tr><td>Water</td><td colspan="4">Tap acidificated water ad libitum</td></tr>
<tr><td rowspan="8">Animal License</td><td colspan="4">BS-1495</td></tr>
<tr><td>Cyno#</td><td>Age</td><td>Weight</td><td>Gender</td></tr>
<tr><td>5548</td><td>12.3</td><td>6.90</td><td>F</td></tr>
<tr><td>5494</td><td>14.3</td><td>5.10</td><td>F</td></tr>
<tr><td>5563</td><td>13.7</td><td>6.20</td><td>F</td></tr>
<tr><td>5554</td><td>7.3</td><td>7.90</td><td>M</td></tr>
<tr><td>5555</td><td>7.3</td><td>8.30</td><td>M</td></tr>
<tr><td>5561</td><td>12.4</td><td>5.30</td><td>F</td></tr>
</table>

Supplementary Table 2. Summary of flow cytometry antibodies and their commercial sources

Target antigen	Clone	Fluorochrome	Supplier
CD20	2H7	PerCP	Biolegend
CD268	11C1	Alexa 647	BD
CD3	SP34-2	APC.Cy7	BD

Supplementary Table 3. Selected antibodies for immunohistochemistry staining

Protein	Manufacturer	Ordering number	Host	Isotype / AntibodyType
CD3	Thermo	9107	Rabbit	IgG / polyclonal
CD3	Roche	790-4341	Rabbit	IgG / polyclonal
CD21	Abcam	ab75985	Rabbit	IgG / polyclonal
Pax5	Abcam	ab109443	Rabbit	IgG / polyclonal
CD8	Abcam	ab4055	Rabbit	IgG / polyclonal
CD1c	Abcam	ab156708	Mouse	IgG1 / monoclonal

Supplementary Table 4. Protocol details of the various antibody treatments used for the immunohistochemistry markers

Antibody	Antigen Retrieval Treatment	Number of retrieval cycles	Retrieval solution	Primary antibody working dilution	Primary antibody incubation time	Secondary antibody	Detection kit
CD3 (Thermo)	Heat	7	CC1	1–50	3 hours	Donkey@ Mouse biotin	DABMap
CD3 (Ventana)	Heat	7	CC1	RTU	32 minutes	Donkey@ Rabbit biotin	DABMap
CD21	Heat	7	CC1	1–100	6 hours	Donkey@ Rabbit biotin	DABMap
Pax5	Heat	7	CC1	1–500	6 hours	Ultramap@ Rabbit HRP	Chromomap
CD8	Heat	7	CC1	1–500	3 hours	Ultramap @ Rabbit HRP	Chromomap
CD1c	Heat	7	CC1	1–100	6 hours	Donkey@ Mouse biotin	DABMap

Supplementary Table 5. Summary of probes used for ISH obtained from Advanced Cell Diagnostics Inc (Hayward, USA).

Probe name	Catalog number	Accession number	Target region
RNAscope® 2.5 VS Probe - Mfa-CD27	486549	XM_005569906.2	319 – 1233
VS Probe - Mfa-PPIB	424149	XM_005559767.1	289 – 1039
Negative Control VS Probe-DapB	312039	EF191515	414 – 862

Supplementary Table 6. Summary of antibodies and their corresponding metal assignments – used for imaging mass cytometry

Antibody	Metal	Clone	Provider
CD3	170Er	Polyclonal	Fluidigm
CD8a	162Dy	D8A8Y	Fluidigm
CD20	167Er	L26	BioSB
CD21	141Pr	EP3093	Abcam
Pax5	164Dy	EPR3720(2)	Abcam
CD163	158Gd	EP324	BioSB
CD68	144Nd	KP-1	BioSB
Vimentin	152Sm	EPR3776	Abcam
Histone H3	176Yb	D1H2	Fluidigm

Supplementary Table 7. List of genes used for single-cell targeted gene expression profiling to identify preexisting B-cell subsets within axillary lymph node populations

Target			
18S	*CD23 (FCER1G)*	*CXCR5*	*IRF8*
ACTB	*CD24*	*E2A (TCF3)*	*ITGa4*
ADA	*CD25*	*EBF1*	*ITGb1*
AICDA (AID)	*CD200*	*FAS (TNFRSF6)*	*CD74*
AURKA	*CD269 (TNFRSF17)*	*FASLG (TNFSF6)*	*MKI67*
BCL2	*CD27 (TNFRSF7)*	*FOXO1*	*MLL (AFF1)*
BCL6	*CD28*	*HDAC4*	*MS4A1*
IGHM	*CD34*	*HDAC5*	*OCT-2 (POU2F2)*
BLIMP1	*CD38*	*HDAC7*	*Pax5*
BLNK	*CD4*	*HDAC9*	*PC1 (CBX2)*
CCNA2	*CD40 (TNFRSF5)*	*ICOSLG*	*POU2AF1*
CCNB1	*CD40LG (TNFSF5)*	*IGHD*	*PCNA*
CCNB2	*CHOP (DDIT3)*	*TFE3*	*PTPRC*
CCR10	*CD5*	*Ikaros (IKZF1)*	*RAG1*
CD10 (MME)	*CD62L (SELL)*	*IL10RA*	*RRM2*
CD11c (ITGAX)	*CD69*	*IL10*	*RUNX1*
CD135 (FLT3)	*CD79a*	*IL11*	*SPIB*
CD138 (SDC1)	*CD79b*	*IL2*	*TGFB1*
CD19	*CD80*	*IL3R*	*TLR2*
CD1c	*CD81*	*IL4*	*TLR4*
CD1d	*CD86*	*IL6*	*TLR7*
CCNE1	*KIT*	*IL7*	*TLR9*
CD21 (CR2)	*CRFL2*	*IL7R*	*TOP2A*
CD22	*CXCR4*	*IRF4*	*XBP1*

The panel includes genes associated with B-cell subsets from previously published signatures, along with relevant genes such as proliferation genes and internal negative controls as well as housekeeping genes.

References for published signatures:

- LeBien TW and Tedder TF (2008). B lymphocytes: how they develop and function. Blood 112, 1570-1580.
- Melchers F (2015). Checkpoints that control B cell development. J Clin Invest 125, 2203-2210.
- Naradikian MS et al. (2014). Understanding B cell biology. In Drugs targeting B-cells in autoimmune diseases, Milestones in drug therapy, X. Bosch et al., eds. (Springer Basel), pp. 11-35.

- R Li et al. (2018). Dynamic EBF1 occupancy directs sequential epigentic and transcriptional events in B-cell programming. Gen Dev 32, 96-111
- T Miyai et al. (2018). Three-step transcriptional priming that drives the commitment of multipotent progenitors toward B cells. Gen Dev 32, 112-126
- P. Engel et al. (2011). Therapeutic targeting of B cells for rheumatic autoimmune diseases. Pharmacol Rev 63, 127-156
- R Nechanitzky et al. (2013). Transcription factor EBF1 is essential for themaintenance of B cell identity and prevention of alternative fates in committed cells. Nature Immunology 14, 867-875

Supplementary Table 8. Overview of variability between individual animals

Marker	Area ratio		No. of follicles	
	Day 21	Day 90 (Termination)	Day 21	Day 90 (Termination)
**CD8+ LN**				
Mean±SD	0.074±0.035	0.150±0.073	4.8±2.6	22.2±13.4
Median	0.074	0.116	5.0	22.0
**CD21+ LN**				
Mean±SD	0.536±0.076	0.702±0.037	9.0±3.4	31.8±17.6
Median	0.516	0.707	9.0	28.0
**CD1c+ LN**				
Mean±SD	0.386±0.204	0.543±0.091	5.8±3.1	21.0±11.1
Median	0.398	0.554	5.0	20
**Dark CD1c+ LN**				
Mean±SD	0.126±0.113	0.201±0.082		
Median	0.105	0.175		
**CD1c+ Spleen**				
Mean±SD		0.213±0.117		
Median		0.217		
**CD3+**				
Mean±SD		0.180±0.069		
Median		0.195		
**CD27+**				
Mean±SD		0.243±0.162		21.2±7.5
Median		0.261		24.0

LN, lymph node; SD, standard deviation

Supplementary Table 9: Proportion of cells expressing a particular gene per cluster

ID	Cluster 1	Cluster 2	Cluster 3	Cluster 4	Cluster 5
ADA	6.7	75.7	74.4	19.1	5.1
AFF1-AF4	66.8	35.9	21.8	71.5	48.9
AICDA	1	74.8	75.6	6.7	1.1
AURKA	0.5	4.9	42.3	1.7	0
BCL2	54.6	6.8	10.3	83.2	19.3
BLNK	87.7	91.3	85.9	93.1	50
CBX2	0	4.9	17.9	0.4	0
CCNA2	2.6	7.8	65.4	4.8	2.8
CCNB1	7.9	51.5	73.1	10.8	4.5
CCNE1	0.5	1.9	33.3	1.2	0
CD19	77.2	98.1	82.1	89.6	39.8
CD1C	86.8	98.1	79.5	90.8	52.3
CD1D	13.2	1.9	2.6	22.4	5.7
CD200	8.2	26.2	44.9	7.7	4
CD210-IL10RA	56.5	47.6	44.9	50.5	7.4
CD22	94.7	99	94.9	94.6	68.2
CD269-BCMA	2.4	36.9	44.9	12.3	8.5
CD27	53.8	98.1	92.3	92.5	63.6
CD275-ICOSLG	88.2	94.2	84.6	65.5	52.3
CD284-TLR4	8.9	54.4	56.4	30.8	10.2
CD29-ITGB1	30.5	88.3	80.8	88.8	31.8
CD38	84.6	13.6	9	82.3	40.3
CD4	8.7	6.8	5.1	10.4	8
CD40-TNFSF5	81	95.1	78.2	85.7	38.1
CD49-ITGA4	80.8	30.1	37.2	82.1	54
CD62L-SELL	87	26.2	24.4	90.6	51.1
CD69	75	28.2	29.5	72.4	16.5
CD80	7.2	30.1	64.1	50.7	13.6
CD81	76.4	94.2	89.7	87.9	43.8
CD86	8.7	49.5	30.8	59	3.4
CD95-FAS	2.6	73.8	69.2	16.4	3.4

CR2	95.9	89.3	76.9	96	66.5
CXCR4	88.7	72.8	80.8	87.1	44.9
CXCR5	93	90.3	70.5	96	51.7
E2A-TCF3	82.7	98.1	98.7	77.8	67
FLT3LG	12.3	14.6	10.3	28.5	10.8
FOXO1	67.1	82.5	84.6	48.7	18.8
HDAC4	28.6	13.6	26.9	22.7	13.6
HDAC5	56	63.1	70.5	52.6	43.8
HDAC9	25.7	67	65.4	36	13.1
IGHD	11.1	2.9	5.1	3.9	2.8
IKFZ1	95	97.1	100	98.1	67.6
IL10	0.2	0	0	0.4	0
IL2RA	23.6	5.8	14.1	16.2	13.1
IL6	9.1	10.7	2.6	28.7	3.4
IL7	7.7	1	3.8	15.4	0.6
IRF4	37.7	15.5	30.8	51.8	21
IRF8	79.1	99	100	92.5	40.9
ITGAX	3.1	4.9	2.6	24.5	5.7
MKI67	1.7	28.2	93.6	2.9	10.2
Pax5	93.5	100	96.2	91.3	69.3
PCNA	88.5	87.4	98.7	91.9	87.5
POU2AF1	75.2	98.1	97.4	93.8	42.6
POU2F2	16.6	55.3	50	18.7	5.7
PRDM1-BLIMP1	61.8	67	51.3	63.4	75
RRM2	15.6	11.7	83.3	26	17
RUNX1-MTG-AML1	33.4	44.7	44.9	27.2	8.5
SPIB	53.1	73.8	69.2	60.7	21
TFE3	4.5	84.5	71.8	74.4	47.2
TGFB1	75	72.8	71.8	85.5	29
TOP2A	78.6	47.6	94.9	78.6	90.3
XBP1	28.1	40.8	56.4	39.1	13.1
CD74	100	100	100	100	100
CD20-MSA4	100	100	100	100	100
BCL6	94.7	100	98.7	91.9	92.6
CD24	91.6	92.2	97.4	91.9	96
DDIT3	93.3	92.2	94.9	89.6	97.2

HDAC7	95.4	98.1	97.4	94.8	93.2
CCNB2	91.3	89.3	97.4	89.6	90.9
CD282-TLR2	95	91.3	97.4	96.5	96.6
CD289-TLR9	99.5	100	97.4	99.4	97.7
TLR7	92.1	92.2	92.3	96.3	96.6
CD45-PTPRC	99.5	100	100	99.8	89.8
CD79A	99.3	100	94.9	99	80.7
CD79B	98.8	100	98.7	98.3	80.7
EBF1	96.2	100	100	96.7	89.2
RAG1	93.5	91.3	94.9	89.4	95.5
